# NF-κB and JAK/STAT Signaling Pathways as Crucial Regulators of Neuroinflammation and Astrocyte Modulation in Spinal Cord Injury

**DOI:** 10.3390/cells13070581

**Published:** 2024-03-26

**Authors:** Tatyana Ageeva, Albert Rizvanov, Yana Mukhamedshina

**Affiliations:** 1 OpenLab Gene and Cell Technology, Institute of Fundamental Medicine and Biology, Kazan Federal University, 420008 Kazan, Russia; rizvanov@gmail.com (A.R.);; 2 Division of Medical and Biological Sciences, Tatarstan Academy of Sciences, 420111 Kazan, Russia; 3 Department of Histology, Cytology and Embryology, Kazan State Medical University, 420012 Kazan, Russia

**Keywords:** astrocyte, glial scar, JAK/STAT, NF-κB, spinal cord injury

## Abstract

Spinal cord injury (SCI) leads to significant functional impairments below the level of the injury, and astrocytes play a crucial role in the pathophysiology of SCI. Astrocytes undergo changes and form a glial scar after SCI, which has traditionally been viewed as a barrier to axonal regeneration and functional recovery. Astrocytes activate intracellular signaling pathways, including nuclear factor κB (NF-κB) and Janus kinase-signal transducers and activators of transcription (JAK/STAT), in response to external stimuli. NF-κB and STAT3 are transcription factors that play a pivotal role in initiating gene expression related to astrogliosis. The JAK/STAT signaling pathway is essential for managing secondary damage and facilitating recovery processes post-SCI: inflammation, glial scar formation, and astrocyte survival. NF-κB activation in astrocytes leads to the production of pro-inflammatory factors by astrocytes. NF-κB and STAT3 signaling pathways are interconnected: NF-κB activation in astrocytes leads to the release of interleukin-6 (IL-6), which interacts with the IL-6 receptor and initiates STAT3 activation. By modulating astrocyte responses, these pathways offer promising avenues for enhancing recovery outcomes, illustrating the crucial need for further investigation into their mechanisms and therapeutic applications in SCI treatment.

## 1. Introduction

Spinal cord injury (SCI) is a critical health issue that arises from damage to the spinal cord, resulting in significant functional impairments below the level of the injury. These impairments can range from loss of mobility to sensory deficits, profoundly affecting the individual’s daily life and requiring comprehensive management strategies to mitigate the impact and enhance recovery potential. The pathophysiology of SCI is multifaceted, involving a cascade of cellular and molecular events. Among the key players in this process are astrocytes, a type of glial cell in the central nervous system (CNS) [1,2,3]. Following SCI, astrocytes undergo a series of changes, culminating in the formation of a glial scar [3,4]. This glial scar has been traditionally viewed as a barrier to axonal regeneration and functional recovery [5].

However, recent insights suggest a more nuanced role of the glial scar and astrocytes in the aftermath of SCI. Astrocytes activate a myriad of intracellular signaling cascades in response to external stimuli. These pathways include the activation of kinases, cyclic AMP (cAMP), and key signaling molecules such as MAP (microtubule-associated protein), nuclear factor κB (NF-κB), and Janus kinase-signal transducers and activators of transcription (JAK/STATs) [6,7,8]. Among the transcription factors, NF-κB and STAT3 play a pivotal role in initiating gene expression related to astrogliosis [9,10]. NF-κB, activated by cytokine receptors and Toll-like receptor 4 (TLR4), targets genes associated with inflammation like tumor necrosis factor-alpha (TNFα) and interleukin-6 (IL-6), amplifying neuroinflammation through microglial activation [9]. STAT3 activation in astrocytes, often initiated by IL-6 released by NF-κB, interacts with the IL-6 receptor [11]. Disrupting STAT3 specifically in astrocytes attenuates astrogliosis across various models of neurodegeneration and trauma. A notable target of STAT3 in this process is the glial fibrillary acidic protein (GFAP), a common marker of astrogliosis [12]. Nonetheless, GFAP’s presence does not directly correlate with the functional outcomes of astrogliosis, indicating the complexity of astrocyte activation and its implications in CNS pathology [12,13]. Signaling pathways are pivotal in shaping the extent to which reactive astrocytes retain, adjust, or inhibit the normal functions carried out by astrocytes in undamaged CNS tissue. They also determine whether reactive astrocytes engage in new activities triggered by injury that could be either advantageous or deleterious. 

The JAK/STAT signaling pathway is a primary route for transmitting signals from cytokines and growth factors [14,15], involved in several biological processes, including leading to the transcription of genes involved in inflammation and glial scar formation [16,17]. In vitro research has shown that astrocytes deficient in STAT3 are more prone to mechanical injuries and exhibit altered structural and metabolic activities. This increased susceptibility is indicated by a decrease in the basal levels of GFAP, lactate dehydrogenase B LDHB, aldolase C (ALDOC), and astrocytic phosphoprotein 15, coupled with an increase in the expression of TPM4 (tropomyosin) and α-actinin 4 [18].

Similarly, the NF-κB pathway, a key regulator of immune and inflammatory responses, is activated in astrocytes following SCI. Activation of NF-κB leads to the transcription of genes associated with inflammation, cell survival, and proliferation. In astrocytes, NF-κB is involved in intricate inflammatory loops [19,20]. It regulates the production and release of pro-inflammatory cytokines like IL-1β, TNFα, and inducible NO synthase (iNOS) [20,21,22,23,24]. In vitro, the dynamic upregulation of GLT-1 in astrocytes, dependent on both the presence of neurons and neuron activity-driven NF-κB activation in astrocytes, is substantially reduced by inhibiting astrocytic NF-κB via the expression of DN-IκB [25].

Understanding the intricate roles of Stat3 and NF-κB signaling in astrocytes and glial scar formation is essential for comprehending the pathophysiology of SCI and could open avenues for novel therapeutic strategies targeting these pathways. This article aims to dissect the roles of these signaling pathways in SCI, shedding light on their potential as therapeutic targets for enhancing functional recovery post-injury.

Here, we systematically consolidated contemporary understandings of the STAT and NF-κB signaling pathways after spinal cord injury. By exploring the potential of targeted interventions in these pathways, we aim to pave the way for innovative therapeutic strategies: pharmacological, cellular, and gene-cellular interventions targeting these pathways for the purpose of modulating astrocyte responses.

## 2. JAK/STAT Signaling Pathway

The JAK/STAT pathway, a critical conduit for signal transduction from extracellular milieu to nuclear gene regulation, encompasses 38 known protein ligands and 36 distinct cell surface receptor combinations [26]. The JAK/STAT signaling pathway incorporates four JAK enzymes: JAK1, JAK2, JAK3, and Tyk2 [27], each integral to transmitting cytokine signals within cells. These kinases are distinguished by their structural domains, including the FERM domain (associated with the proteins four-point-one, ezrin, radixin, and moesin), the Src homology 2 (SH2) domain, a pseudokinase domain, and a kinase domain [26]. The FERM domain has a clover structure composed of F1, F2, and F3 substructures. The SH2 domain also contributes to this receptor binding, while the pseudokinase domain is known to modulate the activity of the kinase domain, ensuring precise control over signaling pathways. Intricately, the structure of these kinases is segmented into seven distinct regions, termed JH1–JH7. JH1, positioned at the C-terminal part of the protein, encodes a kinase domain crucial for the phosphorylation of target substrates. Adjacent to it is JH2, the pseudokinase domain, which, despite lacking enzymatic activity, plays a pivotal role in enhancing the function of the JH1 kinase domain [27]. At the N-terminal end are JH3 to JH7, which collectively contribute to the structural stability and receptor interaction capabilities of the kinase. Specifically, JH5, JH6, and JH7 are instrumental in anchoring the JAK proteins to their corresponding receptors, enabling effective signal transduction.

The STAT family includes seven members: STAT1, STAT2, STAT3, STAT4, STAT5A, STAT5B, and STAT6 [28,29]. Each STAT protein contains an amino-terminal domain, a coiled-coil domain, a DNA-binding domain, an SH2 domain, and a carboxy-terminal transactivation domain [28]. The coiled-coil domain in STAT proteins orchestrates their movement into and out of the nucleus. The SH2 domain of STATs is key in recognizing phosphorylated tyrosine on cytokine receptors [28,30,31]. Thus, the pathway’s activation commences upon ligand–receptor interaction, prompting receptor multimerization and JAK kinase recruitment. JAKs phosphorylate receptor subunits and STAT transcription factors. Phosphorylated STATs then dimerize and translocate to the nucleus to bind specific DNA regulatory sequences and regulate gene expression. 

The JAK/STAT pathway is regulated by suppressors of cytokine signaling (SOCSs), protein inhibitors of activated STATs (PIASs), and protein tyrosine phosphatases [28,31]. SOCSs play a major role in attenuating the JAK/STAT pathway by various mechanisms, including blocking STAT–receptor binding and inactivating JAKs. This regulation is crucial for maintaining cellular homeostasis and preventing the overactivation of the pathway [31]. Additionally, the pathway’s activation can be influenced by non-canonical signals, such as those from receptor tyrosine kinases (RTKs), non-receptor tyrosine kinases (NRTKs), and G protein-coupled receptors.

## 3. JAK/STAT before and after Activation

Under physiological conditions, the presence and distribution of JAK1 and STAT3 are indicative of their integral roles in maintaining CNS homeostasis. JAK1 and STAT3 are not only present within the neuronal cell bodies but also in their processes [32]. This suggests that the JAK/STAT pathway can be activated in situ, even in regions distant from the neuron’s cell body.

Particularly high levels of Jak1 expression are found in the pyramidal neurons of cortical layer V [33]. These neurons, known for their dense projections to the spinal cord, also express STAT3, suggesting a role for the JAK/STAT pathway in corticofugal projection system signaling. It has also been demonstrated that in the intact spinal cord, STAT3 is localized predominantly in motoneurons and dendrite-like structures in the ventral horn [33].

In regions of white matter, such as the corpus callosum and internal capsule, astrocytes demonstrate significant immunoreactivity to JAK1 under physiological conditions [32]. This indicates the JAK/STAT pathway’s role in normal glial cell signaling. Evidence suggests that the JAK/STAT pathway, involving JAK1 and STAT3, is implicated in regulating gliogenesis during neural cell development, particularly in transforming neural progenitors into astrocytes [32,34,35,36].

After SCI, a cascade of molecular events involving the JAK/STAT pathway is initiated. One of the earliest responses is the phosphorylation of JAK1 at Tyr1022/1023 and STAT3 at Tyr705, initiating immediately post-SCI [37,38,39]. This phosphorylation peaks around 12 h post-injury and subsequently decreases. In parallel, the concentration of IL-6 in the injured tissue rises significantly from 3 h post-injury, peaking at the same 12 h mark, and then gradually diminishes. This spike in IL-6 concentration is closely aligned with the phosphorylation events of JAK1 and STAT3 [37,40]. Immunohistochemistry studies have revealed that at 12 h post-SCI, phosphorylated JAK1 and STAT3, along with IL-6, are predominantly expressed in motor neurons within the ventral horns of the spinal cord [37]. Over the long term, phosphorylated STAT3 has been observed in astrocytes and microglia [41,42,43].

Following spinal cord injury (SCI), there is a notable rise in the levels of pro-inflammatory cytokines such as IL-1, IL-6, and TNF-α, both at the mRNA and protein levels [44,45]. These cytokines activate secondary damage mechanisms and are linked to cellular demise after SCI. The initial inflammatory response to SCI, marked by the invasion of neutrophils and the onset of oxidative stress, further triggers the activation of the JAK/STAT signaling pathway [46,47].

Besides IL-6, other members of the IL-6 family cytokines, such as IL-11, ciliary neurotrophic factor (CNTF), leukemia inhibiting factor (LIF), cardiotrophin-1, and oncostatin M, are known to activate the JAK/STAT signaling pathway [48,49,50,51]. These cytokines initiate the dimerization of the gp130 receptor, which then activates JAK. This activation leads to the phosphorylation of STAT3 at Tyr705, facilitating its nuclear translocation [52].

In SCI, the phosphorylation state of STAT3 at Tyr705 persists for up to 168 h following the injury, while IL-6 levels and JAK1 phosphorylation gradually return to baseline. The surge in microglial infiltration, beginning 12 h post-SCI and reaching a peak at 48 h, is proposed to play a significant role in maintaining STAT3 phosphorylation during the chronic phase of the injury [37].

In terms of cellular response, nuclear pSTAT3 is observed in various cell types post-SCI [37,53]. In the acute phase, nuclear pSTAT3 and SOCS3 are detected in neurons and glial cells [53,54]. This activation pattern suggests a significant role for STAT3 in regulating gene expression post-injury, particularly in oligodendrocyte precursor cells (OPCs). The significant involvement of astrocytes in glial scar formation, characterized by the spatial activation of STAT3, will be further discussed in the subsequent section.

## 4. JAK/STAT Pathway in Astrocyte

Research on the JAK/STAT pathway has shown that the stimulation of CNTF receptors in embryonic cells activates JAK1, STAT1, and STAT3, promoting the differentiation of neural stem and neural progenitor cells into astrocytes [55,56]. Studies utilizing conditional knockout mice have demonstrated that a STAT3 deficit significantly reduces astrocyte proliferation and viability, supporting the maintenance of the astrocyte population’s homeostasis [57]. However, excessive expression of STAT3 can lead to pathological conditions in the CNS.

The JAK/STAT3 pathway is crucial in orchestrating astrocyte reactivity, playing a significant role in both acute injuries [58,59] and chronic neurological disorders [57,60]. The activation of this pathway in reactive astrocytes, characterized by STAT3 phosphorylation and increased nuclear localization, is a common response across various CNS diseases, including amyotrophic lateral sclerosis [61], Alzheimer’s disease, and Huntington’s disease [62]. The universal activation of JAK/STAT3 in astrocyte reactivity across different brain disorders and regions underscores its importance in CNS pathology.

In SCI, the JAK/STAT signaling pathway is essential for managing secondary damage following the primary injury. The deletion of STAT3 in astrocytes, particularly those expressing *Nestin* or *GFAP* (genes upregulated in reactive astrocytes), leads to increased lesion areas and reduced glial scar formation, highlighting the pathway’s protective role in glial scar formation [53]. On the other hand, the deletion of SOCS3, a negative regulator, results in prolonged and increased pSTAT3 levels, leading to reduced lesion areas, earlier and more pronounced glial scar formation, and improved motor recovery [63]. Thus, the JAK/STAT pathway is integral in mitigating the extent of injury and facilitating recovery processes post-SCI.

Beyond its role in injury response, the JAK/STAT pathway is essential for astrocyte survival. Astrocytes lacking STAT3 (GFAP:STAT3KO) exhibit increased necrosis and protein release after mechanical injury, indicating the pathway’s role in maintaining astrocyte viability [10]. Moreover, STAT3 regulates the expression of glial filaments, with *GFAP* being a target gene. In GFAP:STAT3KO mice, there is a noticeable reduction in GFAP and vimentin levels [10]. The pathway also modulates astrocyte morphology, particularly in altering cell orientation near injury sites to form the glial scar. In terms of molecular dynamics, research on the activity of STAT-binding elements and the GFAP promoter in the presence of various STAT mutants has revealed that transactivation is dependent on STAT3 activity rather than STAT1 [64]. These studies have shown no synergistic interaction between STAT1 and STAT3, indicating a distinct and critical role for STAT3 in regulating astrocyte responses and functions.

Another transcription factor from the STAT family playing a key role in astrocyte response regulation is STAT5, which is activated by granulocyte-macrophage colony-stimulating factor GM-CSF via the JAK2 pathway [65]. GM-CSF’s role extends to the inhibition of CSPG core protein expression, such as NG2, neurocan, and phosphacan, which are typically induced by TGF-β in primary astrocytes [66]. In this study astrocytes stimulated by TGF-β3, a known inducer of CSPG proteins, demonstrated that GM-CSF not only increases levels of pSTAT5, pAKT, and pRaf but also effectively downregulates TGF-β signaling and CSPG expression [66]. This modulation by GM-CSF is crucial because it suggests a mechanism where STAT5 and PI3K signaling pathways could potentially reduce the secretion of axon-inhibitory proteins by reactive astrocytes, thereby influencing neuroregenerative processes. The ability of JAK and PI3K inhibitors to counteract the effects of GM-CSF further corroborates the regulatory role of these signaling pathways in astrocyte-mediated neuroprotection and regeneration [66,67].

Astrocytes contribute neuroprotectively following SCI through the secretion of cytokines and trophic factors—a process modulated by the JAK/STAT pathway. Reactive astrocytes post-SCI secrete various cytokines, including IL-6, LIF, CNTF, and IL-11, which activate the JAK/STAT pathway, enhancing neurological functions and protecting neurons and oligodendrocytes [68]. The upregulation of these cytokines corresponds with increased JAK1 and STAT3 activation and their nuclear translocation. Notably, inhibiting this pathway with JAK2 inhibitors like AG-490 leads to diminished limb functions post-SCI, emphasizing the protective role of cytokines mediated through the JAK/STAT pathway [69]. Another beneficial outcome of STAT3 activation is its positive impact on the formation of the glial scar, achieved through the inhibition of RhoA. RhoA, a small GTPase, plays a key role in regulating actomyosin tone, cell adhesion turnover, and the migration of reactive astrocytes. By inhibiting RhoA, STAT3 activation facilitates the processes that lead to the formation of a glial scar, which is crucial for the recovery phase following central nervous system injuries [70]. This inhibition, facilitated through the action of ezrin, also affects leukocyte dynamics in vitro. Additionally, reducing phosphatase and tensin homolog (PTEN) levels enhances the dynamics of reactive astrocytes and improves glial scar formation in mice [70,71].

## 5. Nf-κB Signaling Pathway

NF-κB is a pivotal transcription factor family and encompasses proteins from two subfamilies: NF-κB and Rel homologs [72,73]. This family includes p65 (RelA), RelB, c-Rel, NF-κB1 (p105/p50), and NF-κB2 (p100/p52), forming various homo- and heterodimeric complexes [74,75,76]. Each protein contains a highly conserved Rel homology domain (RHD) at the N-terminus, essential for dimerization, DNA binding, and interaction with the IκB family of inhibitory proteins [72,77]. Additionally, the proteins of the Rel subfamily have a transactivation domain at the C-terminus. Precursors p105 and p100 undergo selective ubiquitin-dependent proteasomal degradation, leading to the formation of active p50 and p52 forms, respectively [75,76,77]. Homodimers of p50 and p52 generally act as transcriptional repressors, while combinations including p65, c-Rel, and RelB function as activators [76,77].

NF-κB proteins are defined by their Rel domain and are essential for their nuclear translocation due to the presence of nuclear localization signals (NLSs) [76]. As members of the Rel family, these proteins are equipped with activation domains crucial for initiating gene transcription, and they are distinct in their DNA-binding capabilities, providing an additional level of genetic regulation. In the cytoplasm, NF-κB forms a complex with IκB family proteins, such as IκBα, IκBβ, IκBγ, IκBε, and BCL-3, which are characterized by their ankyrin repeats, crucial for binding NF-κB and keeping it in the cytoplasm [78,79]. This binding mechanism ensures the precise regulation of NF-κB’s activation. The IκB proteins serve as regulatory checkpoints, influenced by various signals like cytokines, viruses, and growth factors, which dictate the activation and nuclear translocation of NF-κB. The precursor molecules p105 and p100 act in a manner similar to IκB proteins by inhibiting NF-κB’s migration to the nucleus, thus playing a pivotal role in its regulation [80]. In inactive states, NF-κB is bound to IκB proteins, preventing its activation and ensuring control over its transcriptional activity until appropriate signaling cues trigger its release and subsequent nuclear entry.

IκB proteins engage with NLSs to promote an alpha-helical structure. IκB-α specifically interacts with the NLS of p65, while IκB-β has the capacity to bind with the NLSs of both p50 and p65 [76]. Such interactions result in a conformation that the nuclear import receptor, importin alpha, does not recognize, in contrast to the random coil structure of IκB-free NLS, which facilitates importin interaction [81]. It has been uncovered that importin α3 predominantly recognizes the NLS of p50 and p65. The dynamic shuttling of the trimeric complex composed of p50, p65, and IκB-α between the nucleus and cytoplasm, alongside a novel route for terminating NF-κB signaling through the promoter-specific degradation of p65 by nuclear proteasomes, underscores the intricacy of NF-κB pathway regulation.

At the onset of signaling, a receptor-bound complex forms, incorporating elements like TRADD, RIP, and TRAF2, laying the groundwork for NF-κB activation [82]. There is also potential for triggering the apoptotic cascade through FADD’s interaction with RIP and TRADD, independent of receptor binding [83,84,85]. TRADD-mediated connections with TRAF1 and TRAF2 contribute to the cell’s defense against apoptosis, notably via cIAP-1 and cIAP-2 proteins, which bolster the TNF-R1 signal against apoptotic signals [85].

NEMO’s role as a regulatory unit within the IKK complex is critical, with its ubiquitination marking an essential step for the activation of the complex. This ubiquitination facilitates the recruitment of IKK to the TNF receptor, ultimately leading to the phosphorylation of IκB-α—a vital phase in the initiation of NF-κB signaling [86,87,88,89,90]. The assembly and activation of the IKK complex are believed to rely significantly on its ability to form multimers. The process is modulated further by the ubiquitination of NEMO and the involvement of deubiquitinating enzymes like CYLD, which are necessary for turning off the IKK complex’s activity [91,92].

The ubiquitination mechanism of the IKK complex influenced various proteins within the cell through the sequential action of enzymes E1, E2, and E3 [93]. Particularly, the SCF (Skp-1/Cul/F-box) type E3 ubiquitin ligase complex, containing the bTrCP subunit, specializes in the poly-ubiquitination of IκB [94].

## 6. Dual Roles of NF-κB in the Nervous System

In the nervous system, the inducible factor NF-κB typically consists of two DNA-binding subunits, such as p50 or p65, which can be either constitutively active or form complexes with the inhibitory subunit IκB-α [95]. Various κB binding activities have been identified, including brain-specific transcription factors (BETA) found in gray matter extracts [86], developing brain factors, and neuronal κB binding factor (NKBF) [96], each with unique binding sequence requirements. These binding activities, not linked to specific genes and not directly tested in reporter gene assays, add an additional layer of complexity to gene expression regulation. Functional NF-κB complexes are present in all cell types of the nervous system, including neurons, astrocytes, microglia, and oligodendrocytes. In neurons, constitutive NF-κB p65 activity, particularly in hippocampal and cortical neurons, results from synaptic activity [97]. This activity is primarily initiated by extracellular Ca^2+^ passing through glutamatergic receptors and L-type Ca^2+^ channels, highlighting the role of NF-κB as a signal transducer from active synapses to the nucleus, involved in maintaining synaptic plasticity [19].

In vitro studies have shown that NF-κB activation protects neurons from amyloid beta peptide toxicity and excitotoxic or oxidative stress [98]. Experiments using adenoviruses to express inhibitors or dominant-negative forms of NIK indicated a reduction in cortical neuron survival, while the overexpression of p65 protected cortical neurons from apoptosis induced by etoposide [99]. Subsequent studies showed that the genetic inhibition of NF-κB in neurons due to an overexpression of the IkB[α] inhibitor reduced neuroprotection following neurotoxic agents’ exposure. Intriguingly, in normal adult brain conditions, NF-κB complexes containing c-Rel are usually undetected [100]. However, activation of metabotropic glutamate receptors can initiate c-Rel’s protective action in neurons, whereas suppression of *c-Rel* genes does not increase cell death without glutamate receptor activation. NF-κB/c-Rel activation promotes neuroprotection by enhancing the transcription of anti-apoptotic genes, particularly relevant in stroke research [101,102]. Conversely, abnormal activation of NF-κB/RelA, characterized by altered acetylation patterns, particularly at Lys 310, can induce the expression of pro-apoptotic genes [103].

It is crucial to note that NF-κB activation in glial cells is generally associated with inflammation. Sustained NF-κB activation in glial cells and neurons up to 72 h is linked to differential use of IκB α and β isoforms, indicating the complexity of NF-κB regulation in the nervous system [95]. The distinct use of IκB α and β isoforms may play a key role in NF-κB regulation [104]. In glial cells, stable presence of active NF-κB following IL-1 stimulation persists even when IκB-α levels return to baseline, while IκB-β concentration remains reduced [104]. This observation suggests that IκB-β may function as a regulator controlling prolonged NF-κB activation. In contrast, a biphasic response suppressed by IκB-α is recorded in cells stimulated by TNF [105]. Signaling pathways in the nervous system leading to NF-κB activation include pathways initiated by receptor activation, such as TNF-α and Fas-ligand [95,106]. Neuron-specific NF-κB stimulating factors, including nerve growth factor (NGF) and the secreted form of beta-amyloid precursor (βAPP), are also found in neurons and glial cells. Central to NF-κB activation is the phosphorylation of IκB by IκB kinase (IKK), consisting of two catalytic subunits (IKK-α and IKK-β) and a regulatory component IKK-γ [24]. Neurons also express mitogen-activated kinase/extracellular signal-regulated kinase 1 (MEKK1), potentially playing a significant role in IKK phosphorylation in response to cell surface receptor activation [107].

Thus, NF-κB can exert both positive and negative influences depending on the cell type and nature of damage. Traumatic brain injury, excitotoxicity, and ischemia are characterized by NF-κB activation. Simultaneously, basal NF-κB activity in neurons is necessary for protecting these cells from traumatic damage, with the effectiveness of protective effects depending on the timing post-injury. Endogenous NF-κB activation might protect neurons under stress conditions caused by damage by suppressing p53-mediated apoptosis through the internal mitochondrial pathway. Additionally, the protective role of NF-κB in neurons includes anti-apoptotic effects mediated by induction of caspase inhibitors or expression of antioxidant genes. In contrast, inhibiting NF-κB in microglia and astrocytes may contribute to favorable outcomes following CNS injury.

## 7. NF-κB Pathway in Astroglios

**Figure 1:**
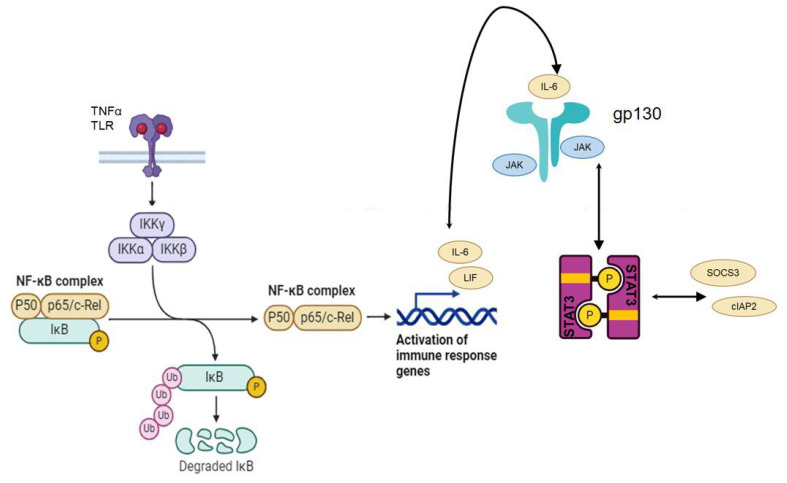
The interaction between the nuclear factor κB (NF-κB) and signal transducer and activator of transcription 3 (STAT3) pathways, particularly in response to TNF-α stimulation, involves a complex cascade of events. Activation of NF-κB leads to the transcription of certain genes, including interleukin-6 (*IL-6*) and leukemia inhibitory factor (*LIF*). The IL-6 produced by this process can act in an autocrine or paracrine manner, activating its receptor and leading to further signaling events. This activation involves the IL-6R/glycoprotein (gp130) complex and Janus 1/2 (JAK1/2) kinases, which then phosphorylate STAT3. Phosphorylated STAT3 enters the nucleus to initiate transcription of its target genes, including suppressor of cytokine signaling 3 (*SOCS3*) and cellular inhibitor of apoptosis 2 (*cIAP2*). This cycle illustrates the intricate crosstalk between these two pathways in cellular signaling.

Astrocytes demonstrate significant NF-κB activity, highlighting their role in NF-κB signaling. IkBa, an NF-κB downstream target, is more prominently expressed in astroglia than neurons, indicating astroglia as the primary site for IkBa expression and NF-κB activity [108]. The selective inhibition of astroglial NF-κB signaling using GFAP-IkBα-dn in a vascular dementia mouse model resulted in reduced gliosis and axonal loss, improved white matter integrity and memory function, and decreased microglial activation and demyelination [109]. TNFα significantly upregulates IkBa in astroglia, underscoring astroglia’s role in NF-κB signaling (Figure 1) [110].

**Figure 2:**
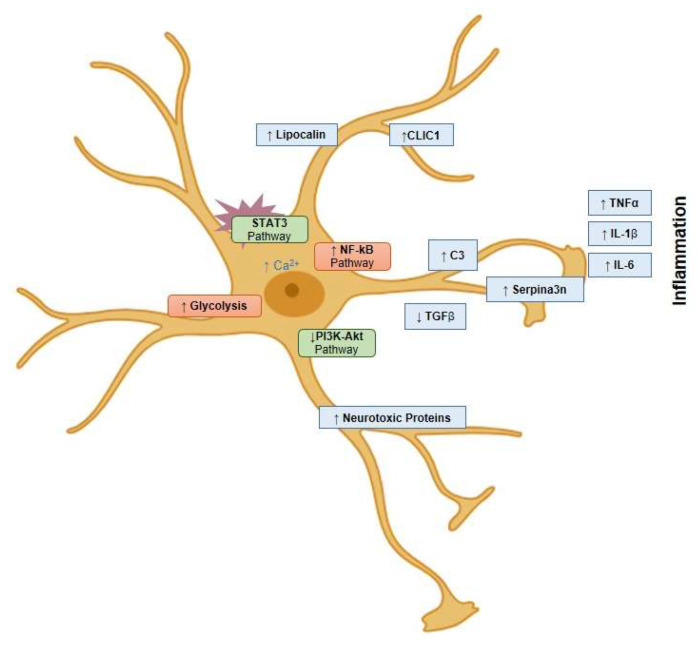
In response to damage, astrocytes are characterized by the activation of inflammatory pathways, including NF-κB. This leads to increased expression of components like complement component expression (C1r, C1s, C3, and C4), contributing to synaptic damage. They also express inflammatory mediators such as IL-1β, tumour necrosis factor α (TNF-α), and IL-6. These astrocytes exhibit hyperphosphorylation and activation of the STAT pathway, typically in response to injury. Additionally, the inhibition of the anti-inflammatory phosphoinositide 3-kinases (PI3K-Akt) pathway in these cells results in decreased expression of neurotrophic factors, which contributes to synaptic loss and apoptosis.

TNFα can convert primary astrocytes into immature precursors, signifying NF-κB’s key role in astrocyte differentiation [95]. This process involves changes in markers like CD44 and CD133 [111]. TNFα treatment suppresses astrocytic markers such as GFAP and ALDH1A1, affecting astrocyte differentiation in inflammatory states [112]. NF-κB activation in astrocytes prompts C3 protein release, disrupting neuronal dendritic morphology and network function (Figure 2) [108]. This suggests that astrocytic NF-κB activation directs differentiation towards a reactive gliotic phenotype.

NF-κB activation in astrocytes not only modulates their fate but also may facilitate their conversion into neurons by reducing Hes1 and increasing Hes5 expression [113]. This aligns with studies showing that astrocytes can be reprogrammed into neurons post-injury. Using JSH-23 to block the NF-κB pathway inhibits the neutrosphere’s differentiation into astrocytes, inducing apoptosis. RELA/p65 acts as the primary effector against apoptosis during the first 24 h of astrocyte differentiation [111]. During the glutamatergic differentiation of neural crest stem cells, nuclear p65 levels decrease while c-REL increases. NF-κB plays a dual role as both an attenuator and promoter of apoptosis. 

Astrocyte tolerance to RelB is noteworthy. When RelB is synthesized and phosphorylated at Ser472, it remains bound to DNA, maintaining RelB/p50 stability on cytokine promoters. This differs from mechanisms in other cell types like microglia and monocytes [114]. Astrocytic tolerance occurs as p65/p50 dimers are removed from DNA by newly synthesized IκBα entering the nucleus, and then they are replaced with RelB/p50 complexes [111]. The phosphorylation of RelB Ser472 prevents the removal of RelB/p50 complexes from DNA, sustaining their activity on cytokine promoters. Additionally, RelB’s interaction with SIRT1 may facilitate p65 deacetylation and removal from DNA [111,115]. Unlike microglia, no epigenetic changes in cytokine promoters regulated by *RelB* are observed in astrocytes. Astrocytic RelB also regulated tolerance in vivo in a mouse model of systemically induced neuroinflammation.

## 8. NF-κB/STAT3 Signaling Crosstalk

The interaction and linkage between JAK/STAT signaling pathways, particularly STAT3 and NF-κB in nervous tissue and specifically in astrocytes, are not well understood. In vitro studies using a neurosphere model have shown that NF-κB activation suppresses the STAT3 pathway 48 h into cell differentiation, a period when signaling is typically activated. This process involves STAT3 signaling inducing astrocyte differentiation through the activation of astrocyte-specific genes like *GFAP*. This study also revealed that *GFAP* expression was reduced at 48 h, 72 h, and 1 week following TNF treatment compared to untreated cells [111].

In certain cell types, like cancer cells, constitutively activated STAT3 causes the hyperacetylation of RelA, a subunit of NF-κB, through interactions with p300 [29]. This action prolongs NF-κB’s nuclear retention, enhancing its activation. NF-κB, in turn, can activate STAT3 through cytokines such as IL-17, IL-21, and IL-23 [116]. Interestingly, STAT6, activated by IL-4, acts as an NF-κB antagonist, particularly at the E-selectin gene promoter, revealing a nuanced regulatory interplay [117].

NF-κB collaborates with other transcription factors like Sp1, AP-1, and CEBP/β. Sp1, located near κB sites, jointly induces genes with NF-κB, such as *ICAM-1* and *GM-CSF*. The AP-1 factors (Fos and Jun proteins) also interact with NF-κB, and they mutually enhance each other’s transcriptional activity [118]. Additionally, CEBP/β and NF-κB demonstrate synergism, with their ratio determining the outcome of gene expression.

Post-injury, cytokines activate the JAK/STAT pathway, leading to the transcriptional regulation of target genes. JAK kinase also activates the PI3K/Akt pathway [119]. Phosphorylated Akt then activates IκB kinase, promoting NF-κB activation and the subsequent transcription of target genes [87]. In this context, NF-κB plays a crucial role in cell fate decisions during astrocyte specification, particularly in inhibiting apoptosis and influencing the response to various developmental signals like NOTCH and JAK/STAT.

NF-κB is known for its pro-inflammatory role, whereas pathways like PI3K-Akt can have anti-inflammatory effects [119]. The balance between these pathways influences cellular responses such as astrocyte polarization, impacting inflammatory states and cell proliferation.

## 9. Conclusions

The study of NF-κB and JAK/STAT pathways in the context of SCI reveals a complex network of signaling events crucial for understanding neuroinflammation and glial dynamics. NF-κB’s role extends beyond its traditional pro-inflammatory capacity, intricately interacting with various cellular processes and other signaling pathways, including JAK/STAT. This interaction is pivotal in determining the fate of astrocytes post-SCI, influencing their potential to both harm and repair neural tissue. Research involving rodent models and preliminary human studies indicates the potential benefits of engaging intact fibers in the healing process. This could be achieved by encouraging the sustained activation of STAT3 and/or the suppression of NF-κB, pointing to the promising effects of targeting receptors and molecules that are involved in the activation or inhibition of both pathways simultaneously. These therapeutic strategies foster prospects for advanced research dedicated to modulating these signaling networks, which could catalyze the development of innovative therapeutic strategies for spinal cord injuries and various neurological disorders.
